# Idiopathic Capital Femoral Chondrolysis: A Case Report

**DOI:** 10.7759/cureus.28789

**Published:** 2022-09-05

**Authors:** Pranav Gupta, Dhananjay Gupta, Sandeep Shrivastav

**Affiliations:** 1 Orthopaedics, Jawaharlal Nehru Medical College, Datta Meghe Institute of Medical Sciences, Wardha, IND; 2 Orthopaedics, Fortis Flt. Lt. Rajan Dhall Hospital, Vasant Kunj, New Delhi, IND

**Keywords:** limping, hip stiffness, abduction and external rotation contracture, painless limping, paediatric, hip joint, idiopathic, chondrolysis, case report

## Abstract

Idiopathic capital femoral chondrolysis is a rare condition most commonly seen in African-American pre-adolescent females. The primary symptoms are hip stiffness and pain, which are accompanied by limping. Physical examinations typically reveal an external rotation contracture, flexion contracture, and abduction contracture. There is also immobility at the hip secondary to muscle spasms. Here, we present and discuss the follow-up case of a 10-year-old female patient who was diagnosed with idiopathic chondrolysis of capital femoral epiphysis, right side, and underwent right hip arthroscopic evaluation and lavage. At follow-up two months after the right hip arthroscopic evaluation, she presented with painless limping on the right side. The patient underwent soft tissue release right hip under general anaesthesia and was discharged in stable condition.

## Introduction

In 1930, Waldenström first described chondrolysis of the hip joint [1]. It is a well-known repercussion of slipped capital femoral epiphysis [2-4]. It can also be caused by trauma [5,6], infective arthritis [7], prolonged immobilisation [7,8], Stickler syndrome, Marfan's syndrome, and juvenile idiopathic arthritis [1,6,7]. Females are affected more commonly than males, the ratio being 6:1 [8]. Latest demographic studies reveal an equitable geographical distribution of the condition, which was first believed only to affect teenage African American females [5,9,10]. Although it can be bilateral, involvement is most commonly unilateral.

Idiopathic capital femoral chondrolysis is characterised by rapidly accelerating articular cartilage damage in the hip joint leading to premature degeneration and successive joint arthrosis. The majority of patients complain of the hip, knee, or inguinal pain, along with rigidity or limping in the pathological hip. Lab investigations are routinely normal. The characteristic radiographic result is a concentric narrowing (lower than 3 millimetres) of the joint space along with periarticular osteopenia [11-13]. Magnetic resonance imaging (MRI) presents characteristic findings of focal or global chondral oedema with cartilage thinning initially. It is difficult to foresee how the disease will take its course, with or without therapy. In this report, we present and discuss the case of a 10-year-old female patient diagnosed with rare right-sided idiopathic capital femoral chondrolysis and its management.

## Case presentation

A female patient, aged 10, presented at the orthopaedics outpatient department with the chief complaints of gradually increasing pain in the right hip since three months, resulting in difficulty bearing weight. The pain radiated up to the right knee and was present at rest and during movement. The patient was admitted for pain management and investigations. Physical examination revealed all her vitals being within the normal ranges. The patient was conscious, cooperative, and well oriented to time, person, and place. Her body temperature was 98.6°F, heart rate 82 per minute, respiratory rate 18 cycles per minute, oxygen saturation 98% on room air, and blood pressure 110/70 mm of Hg. No pallor, icterus, clubbing, cyanosis, oedema, or lymphadenopathy were present. All the systemic examinations were normal.

**Figure 1:**
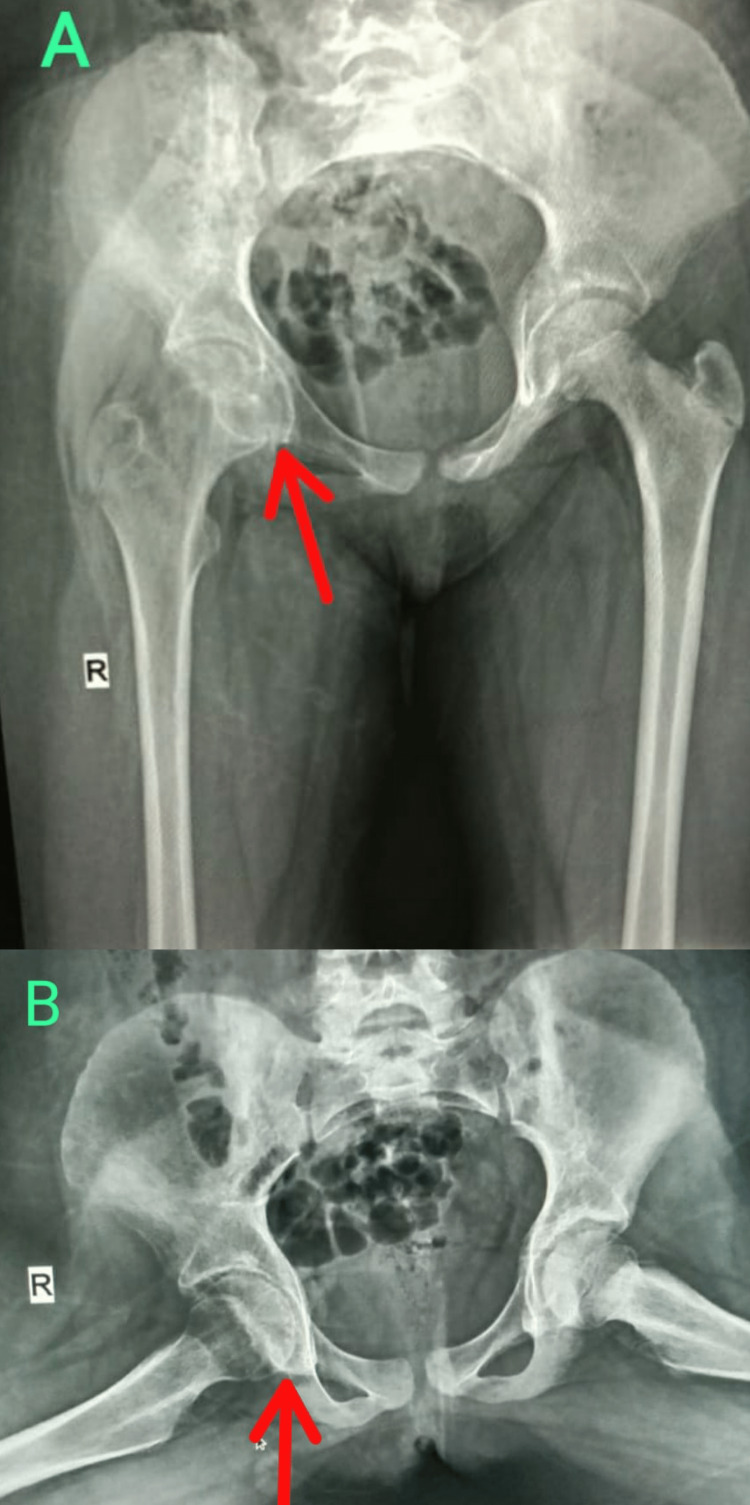
Digital skiagram (hips) at the time of presentation (A) anteroposterior view showing mild pelvic tilt with asymmetry of the pelvis, and mildly concentric narrowing of the joint space at the right hip joint (red arrows); (B) lateral (frog) view showing minimal irregularity along the inferior medial margin of the right femoral head (red arrows).

**Figure 2:**
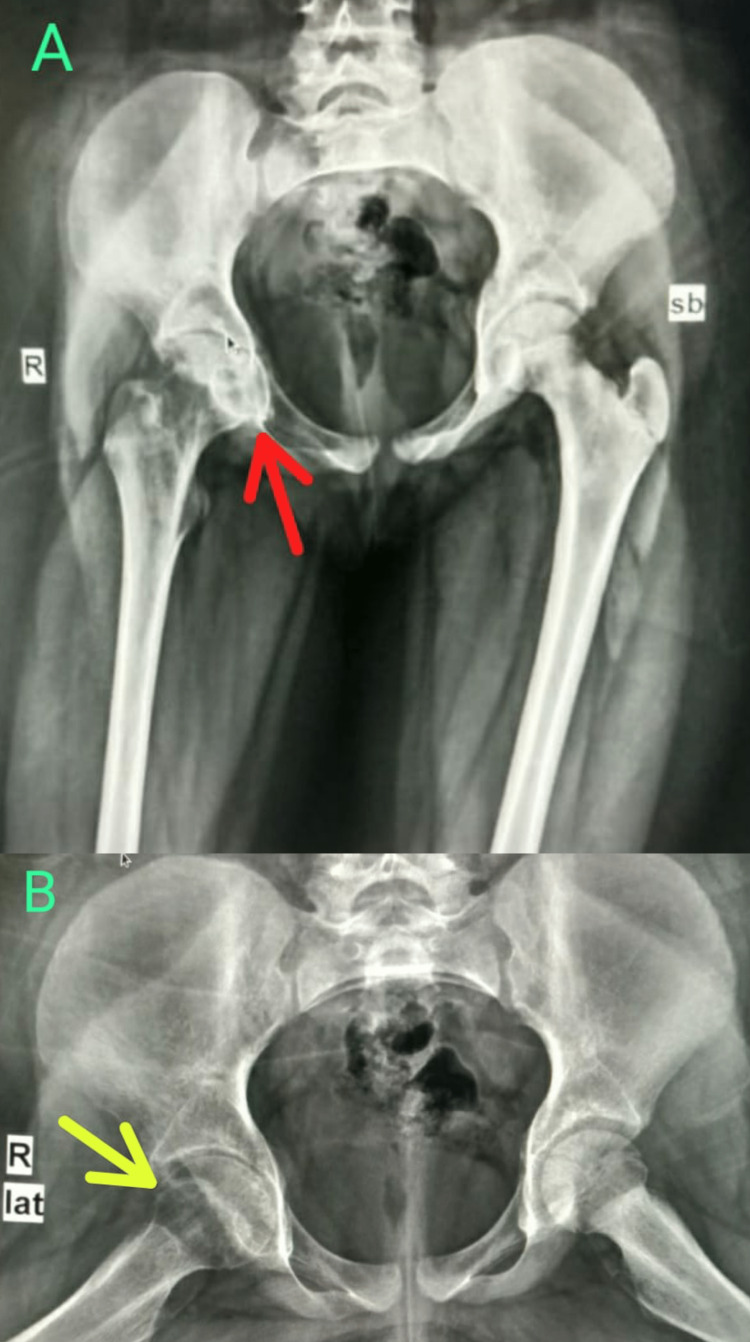
Digital skiagram (hips) at two months after presentation (A) anteroposterior view showing mild pelvic tilt with asymmetry of pelvis, mildly concentric narrowing of the joint space, and periarticular osteopenia at the right hip joint (red arrow); (B) lateral (frog) view showing few subarticular lucencies are seen in right femoral head (yellow arrow).

**Figure 3:**
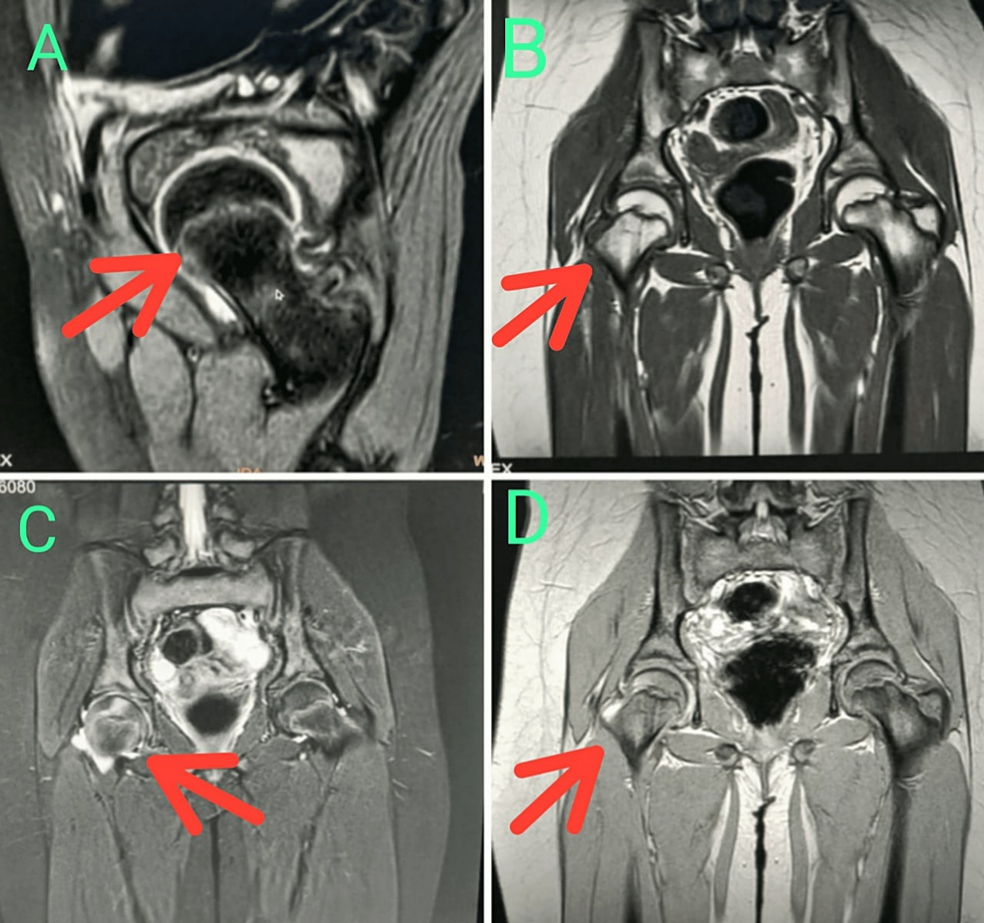
MRI of the hip regions at the time of presentation. (A, B) T1 weighted, (C, D) T2 weighted Focal marrow oedema (red arrows) in the right femoral epiphysis with minimal joint effusion. Possibility of idiopathic chondrolysis of hip.

Upon right hip examination, there was generalized wasting of the glutei and quadriceps muscles along with painful limitation of hip joint movements. There was an attitude of 15° flexion, 30° abduction, and 20° external rotation at the hip. There was no distal neurovascular deficit. Her laboratory reports were normal, with only the erythrocyte sedimentation rate (ESR) being raised (Table 1). On imaging, her pelvic X-ray showed mild pelvic tilt, pelvic asymmetry, and mildly reduced joint space at the right hip (Figures 1, 2). MRI findings were focal marrow oedema in the right femoral epiphysis with minimal joint effusion and minimal cartilage thinning (Figure 3). A diagnosis of primary idiopathic capital femoral chondrolysis was made. For confirmation, it was decided to do hip arthroscopy and take biopsy from any abnormal tissue, especially synovium. The patient was operated on after obtaining informed consent and anaesthetic clearance. The patient underwent right hip arthroscopic evaluation under general anaesthesia. There was no intra-articular pathology found either in synovial tissue or femoral and acetabulum cartilage. Hence, the diagnosis was confirmed. The joint was irrigated with 3 litres of normal saline. The patient tolerated the procedure well. The postoperative period was uneventful, and the dressing was changed on the first postoperative day. The patient was kept on skin traction. Further treatment modalities like immunomodulation, disease-modifying antirheumatic drugs (DMARDs), and biologicals were discussed with rheumatologist and paediatric teams, and it was decided to put the patient under observation. The patient was discharged in stable condition. She was instructed to take the skin traction off three times a day for range of movement exercises for the hip and knee joints. The total duration of skin traction was three weeks to keep the patient non-weight bearing.

**Table 1:** Laboratory investigations at the time of presentation All indices are within the normal limits except for ESR, which is 24 mm at one hour, higher than the normal range.

Test Report	Result	Normal Value	Unit
Blood Count			
Haemoglobin	12.7	11.5 - 15.5	gm/dL
Red Blood Cell (RBC)	4.59	4.0 - 5.2	million/μL
White Blood Cell (WBC)	7.68	5.0 - 13.0	thousand/μL
Platelet	276	170 - 450	thousand/μL
RBC and Platelet Indices			
Hematocrit	37.16	35 - 45	%
Mean Corpuscular Volume	81.0	77.0 - 95.0	fL
Mean Corpuscular Haemoglobin Concentration	34.2	31.0 - 37.0	g/dL
Mean Corpuscular Haemoglobin	27.7	25.0 - 33.0	pg
Red Cell Distribution Width	14.7 (High)	11.6 - 14.0	%
Mean Platelet Volume	11.1 (High)	6.8 - 10.9	fL
WBC Differential Count			
Segmented Neutrophils	60	37 - 65	%
Absolute Neutrophil Count	4.61	2.0 - 8.0	thousand/μL
Lymphocytes	33	28 - 48	%
Absolute Lymphocytes Count	2.53	1.0 - 5.0	thousand/μl
Neutrophil Lymphocyte Ratio	1.8	0.78 - 3.53	
Erythrocyte Sedimentation Rate (ESR)	24 (High)	0 - 10	mm at 1 hour

The patient followed up after two months of right hip arthroscopic evaluation for primary idiopathic chondrolysis of right hip and presented with painless limping towards the right side. The patient was admitted, and her physical examination was done; all her vitals were within the normal range. The patient was conscious, cooperative, and well oriented to time, person and place. Her body temperature was 98.6°F, heart rate 102 per minute, respiratory rate 20 cycles per minute, oxygen saturation 98% on room air, and blood pressure 110/70 mm of Hg. All the systemic examinations were normal.

**Figure 4:**
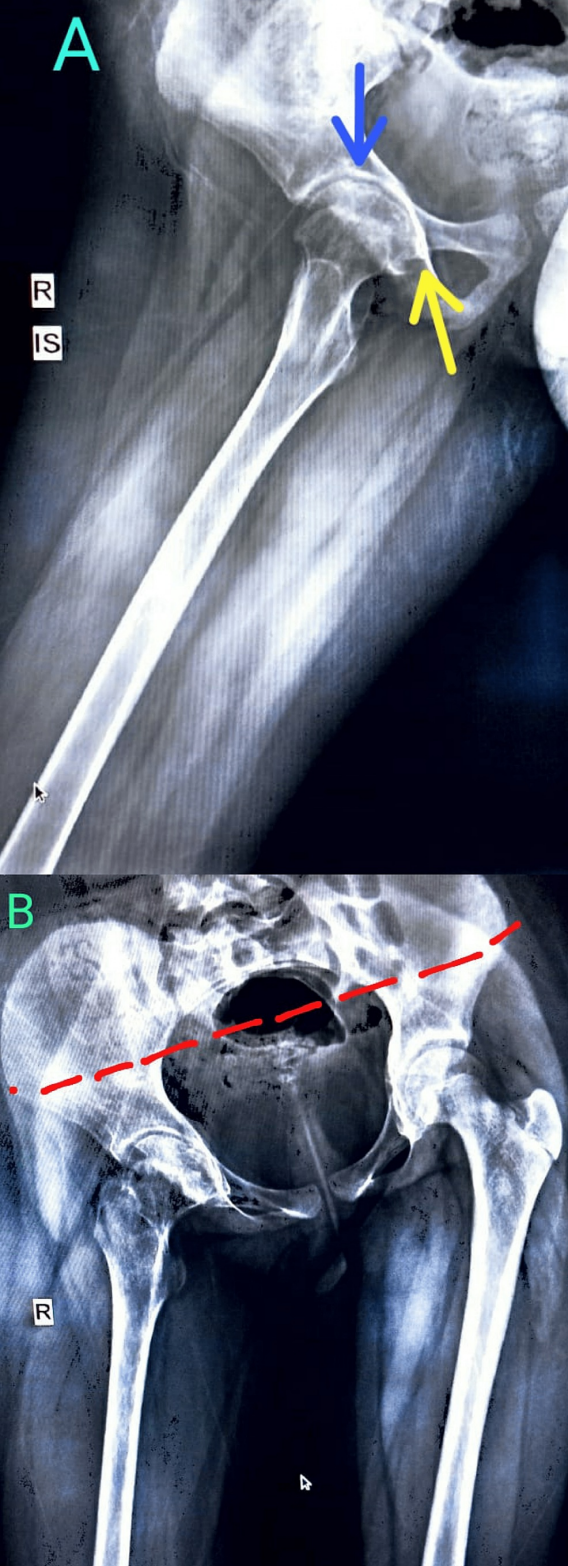
Digital skiagram (hips) at follow-up after arthroscopic evaluation. (A) lateral view, (B) anteroposterior view There is a pelvic tilt (red line) towards the right side. There is mild flattening of right femoral head with minimal sclerotic margin (blue arrow). Rest of the metadiaphysis appears normal.

Upon right hip examination, there was generalized wasting of the glutei and quadriceps muscles along with fixed deformity of flexion, abduction, and external rotation contracture at the hip. Her pelvic X-ray was done with the findings denoting pelvic tilt towards right side and mild flattening of right femoral head with minimal sclerotic margin (Figure 4). Based on the physical examination, right hip examination, and diagnostic imaging, the team came to the diagnosis of right hip abduction and external rotation contracture. The patient was operated on after informed consent and anaesthetic clearance. The patient underwent soft tissue release of right hip under general anaesthesia. The patient was positioned in lateral position, and an incision was given as per posterolateral approach to the hip. The tight, thickened fascia over the glutei muscles was divided along with the iliotibial (IT) band. Contraction was confirmed on the table by achieving full adduction and internal rotation. The postoperative period was uneventful, and the dressing was changed on the first postoperative day. The patient was ambulatory and discharged in stable condition. No skin traction was given. The patient was allowed weight bearing as per pain tolerance and range of movement exercises.

**Figure 5:**
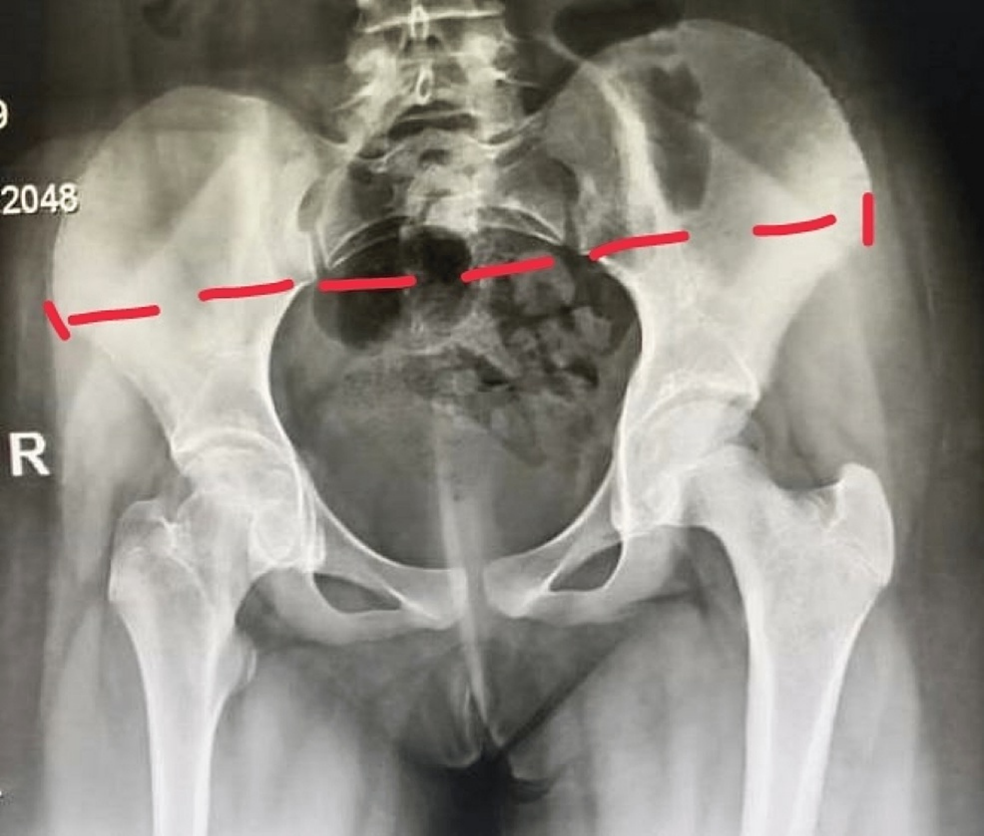
Digital skiagram (hips) AP view at follow-up after soft tissue release There is correction in the pelvic tilt (red line) observed and no other deformity identified. AP: anteroposterior

The patient followed up two months after soft tissue release of right hip and presented without any pain or limping with full mobility at the right hip joint. Her pelvic X-ray was done where correction in the pelvic tilt was observed and no other deformity was identified (Figure 5).

## Discussion

Idiopathic chondrolysis of the hip has no specific diagnostic criteria. The typical presentation is of a young adolescent female with clinical manifestations such as hip stiffness and pain with concurrent limping. Typically, flexion, abduction, and external rotation contractures are visible on physical examination. The hip's range of motion may be severely restricted as a result of muscle spasms and fibrous ankylosis. Diagnosis of the disease is made by ruling out septic arthritis, juvenile idiopathic arthritis, pigmented villonodular synovitis, tuberculosis hip, Perthes disease, slipped capital femoral epiphysis, and uncommon tumours like osteoid osteoma, which are the possible differential diagnosis. 

The patient has normal laboratory findings with slightly elevated ESR. The distinctive radiological features of the disease include periarticular osteopenia in addition to concentric narrowing (less than 3 millimetres) of the joint spaces [11-13]. Pain relief, deformity correction, and motion restoration are the three objectives of treatment [12]. In this case report, the patient presented with clinical features imitating infectious arthritis and juvenile idiopathic arthritis. However, the characteristic radiographic findings of idiopathic capital femoral chondrolysis were reported.

The physical exam, right hip examination, and diagnostic imaging all directed to the diagnosis of idiopathic capital femoral chondrolysis. The patient underwent surgery to confirm the diagnosis and rectify the anomaly. The patient was pain-free on the follow-up but complained of painless limping towards the right side. She was diagnosed with right hip abduction and external rotation contracture and was operated on for the same. The outcome for the patient was satisfying as she presented without any pain or limping, and with full mobility at the hip joint upon follow-up after the procedure.

For early diagnosis of idiopathic capital femoral chondrolysis, spreading awareness about its occurrence is vital as it can be misinterpreted as chronic septic or inflammatory arthritis. The most common complications of the discussed disease are protrusio acetabuli, end-stage arthritis, and spontaneous hip fusion. More studies are needed on the aetiology and pathophysiology of idiopathic chondrolysis of the hip that can result in more effective management for better patient outcomes.

## Conclusions

Hip joint chondrolysis in children is a unique but severe condition characterised by hip pain, stiffness, and concentric radiological narrowing of joint space. A previously healthy hip joint may develop ankylosis due to the unpredictability of its clinical course. If contractures are present, performing an early examination while under anaesthesia is advised before performing an open soft tissue release. A thorough followup is essential. The patient followed up two months after soft tissue release of right hip and presented without any pain or limping with full mobility at the right hip joint. In our case, early diagnosis, prompt treatment, and close patient followup led to a satisfactory outcome for the patient.
